# A review of current therapeutics targeting the mitochondrial protease ClpP in diffuse midline glioma, H3 K27-altered

**DOI:** 10.1093/neuonc/noad144

**Published:** 2023-08-17

**Authors:** Evangeline R Jackson, Mika L Persson, Cameron J Fish, Izac J Findlay, Sabine Mueller, Javad Nazarian, Esther Hulleman, Jasper van der Lugt, Ryan J Duchatel, Matthew D Dun

**Affiliations:** Cancer Signalling Research Group, School of Biomedical Sciences and Pharmacy, College of Health, Medicine and Wellbeing, University of Newcastle, Callaghan, New South Wales, Australia; Precision Medicine Research Program, Hunter Medical Research Institute, New Lambton Heights, New South Wales , Australia; Cancer Signalling Research Group, School of Biomedical Sciences and Pharmacy, College of Health, Medicine and Wellbeing, University of Newcastle, Callaghan, New South Wales, Australia; Precision Medicine Research Program, Hunter Medical Research Institute, New Lambton Heights, New South Wales , Australia; Cancer Signalling Research Group, School of Biomedical Sciences and Pharmacy, College of Health, Medicine and Wellbeing, University of Newcastle, Callaghan, New South Wales, Australia; Precision Medicine Research Program, Hunter Medical Research Institute, New Lambton Heights, New South Wales , Australia; Cancer Signalling Research Group, School of Biomedical Sciences and Pharmacy, College of Health, Medicine and Wellbeing, University of Newcastle, Callaghan, New South Wales, Australia; Precision Medicine Research Program, Hunter Medical Research Institute, New Lambton Heights, New South Wales , Australia; DIPG/DMG Center Zurich, University Children’s Hospital Zürich, Zurich, Switzerland; Department of Neurology, Neurosurgery and Pediatric, UCSF, San Francisco, California, USA; DIPG/DMG Center Zurich, University Children’s Hospital Zürich, Zurich, Switzerland; Center for Genetic Medicine Research, Children’s National Hospital, Washington, District of Columbia, USA; The George Washington University, School of Medicine and Health Sciences, Washington, District of Columbia, USA; Princess Máxima Center for Pediatric Oncology, Utrecht, Netherlands, Utrecht, Netherlands; Princess Máxima Center for Pediatric Oncology, Utrecht, Netherlands, Utrecht, Netherlands; Cancer Signalling Research Group, School of Biomedical Sciences and Pharmacy, College of Health, Medicine and Wellbeing, University of Newcastle, Callaghan, New South Wales, Australia; Precision Medicine Research Program, Hunter Medical Research Institute, New Lambton Heights, New South Wales , Australia; Cancer Signalling Research Group, School of Biomedical Sciences and Pharmacy, College of Health, Medicine and Wellbeing, University of Newcastle, Callaghan, New South Wales, Australia; Precision Medicine Research Program, Hunter Medical Research Institute, New Lambton Heights, New South Wales , Australia; Paediatric Program, Mark Hughes Foundation Centre for Brain Cancer Research, College of Health, Medicine and Wellbeing, University of Newcastle, Callaghan, New South Wales, Australia

**Keywords:** CLPP, CLPP agonist, DMG, dordaviprone, ONC201

## Abstract

Diffuse midline gliomas (DMGs) are devastating pediatric brain tumors recognized as the leading cause of cancer-related death in children. DMGs are high-grade gliomas (HGGs) diagnosed along the brain’s midline. Euchromatin is the hallmark feature of DMG, caused by global hypomethylation of H3K27 either through point mutations in histone H3 genes (H3K27M), or by overexpression of the enhancer of zeste homolog inhibitory protein. In a clinical trial for adults with progressive HGGs, a 22-year-old patient with a thalamic DMG, H3 K27-altered, showed a remarkable clinical and radiological response to dordaviprone (ONC201). This response in an H3 K27-altered HGG patient, coupled with the lack of response of patients harboring wildtype-H3 tumors, has increased the clinical interest in dordaviprone for the treatment of DMG. Additional reports of clinical benefit have emerged, but research defining mechanisms of action (MOA) fall behind dordaviprone’s clinical use, with biomarkers of response unresolved. Here, we summarize dordaviprone’s safety, interrogate its preclinical MOA identifying the mitochondrial protease “ClpP” as a biomarker of response, and discuss other ClpP agonists, expanding the arsenal of potential weapons in the fight against DMG. Finally, we discuss combination strategies including ClpP agonists, and their immunomodulatory effects suggestive of a role for the tumor microenvironment in DMG patient response.

**Key Points**

- Dordaviprone shows emerging clinical benefits for the treatment of diffuse midline glioma (DMG).
- In DMG, dordaviprone is an agonist of the mitochondrial protease ClpP.
- *CLPP* is overexpressed in DMG patients, highlighting the therapeutic potential of ClpP agonists.

Diffuse midline glioma (DMG), including those that arise in the pontine region of the brainstem (referred to as diffuse intrinsic pontine glioma [DIPG]), is the most aggressive and lethal childhood cancer, harboring a median overall survival (OS) of 9–11 months, with a 2-year OS of <10%.^1,2^ Over 60 years of clinical trials testing cytotoxic chemotherapies, chemoradiation, precision- and immuno-therapies, surgical resection, and local therapeutic delivery in the treatment of DMG have not improved patient outcomes.^3^

In 2021, The World Health Organization (WHO) published the fifth classification of central nervous system (CNS) tumors and reclassified DIPG as “diffuse midline glioma, H3 K27-altered,” representing high-grade gliomas (HGG) located along the midline structures of the brain.^4^ The new classification recognized global hypomethylation of lysine 27 of histone H3 (H3K27) as a ubiquitous feature of DMG, driving epigenetic signatures that promote promiscuous expression of oncogenes resulting in rapid tumor growth,^3,5^ as well as imparting immune system privilege.^6^

Remarkably, 80% of DMG cases harbor missense mutations at lysine 27 in histone H3 in either *HIST1H3B/C* (H3.1K27M) or *H3F3A* (H3.3K27M) genes, where the amino acid lysine is substituted to a methionine.^7,8^ Patients carrying wildtype-H3 overexpress the *e*nhancer of *z*este *h*omolog *i*nhibitory *p*rotein (EZHIP) which harbors biochemical properties similar to the H3K27M mutation, both of which drive hypomethylation of H3K27.^2,9^

There are subtle differences in the median OS of individual DMG patients harboring different H3-alterations and tumor localizations, that is, pons compared with thalamus.^2^ Indeed, patients with H3.1K27M mutations are most frequently diagnosed with tumors in the pontine region of the brainstem at a median age of 4 years old and harbor a median OS of 12–15 months.^2^ Whereas, H3.3K27M alterations are frequently diagnosed in the pons, but are also seen in the spine, midbrain, and thalamus with patients diagnosed at a median age of 6–7 years of again with OS of 9 months.^2,10^ Lastly, DMG patients carrying wildtype H3K27 but overexpression of EZHIP, are diagnosed with tumors evenly distributed along the midline and diagnosed at a median age of 4.6 years of age, with slightly increased OS of 15 months compared with H3.1K27M and H3.3K27M patients.^2^

During development, the functional organization of chromatin is regulated by the polycomb repressive complex 2 (PRC2), particularly, by the catalytic enzyme of the lysine methyltransferase EZH2 responsible for the deposition of trimethylated marks (me3) on histone tails, specifically at H3K27.^3^ EZH2 directly regulates chromatin accessibility and the transcriptional efficiency/activity of the developing midline.^3^ Although H3 mutations are heterozygous and only contribute to a fraction of the total pool of H3 protein (3%–17%), its effects are global,^7^ promoting dominant negative H3K27 trimethylation (H3K27me3).^7,11,12^ H3 proteins carrying the mutant methionine (H3K27M) sequester the EZH2 methyltransferases through interactions with its C-terminal SET domain^13^ to reduce the global activity of the PRC2 complex.^10,14^ This, coupled with the co-enrichment of elevated H3K36me2 deposited by the nuclear receptor binding SET domain protein 1/2 (NSD1/2),^15,16^ inhibit spreading of repressive H3K27me3 marks that are preferentially retained at unmethylated CpG islands, affecting lowly expressed genes influencing neurogenesis.^8,11,17–19^ Global loss of H3K27me3 promotes H3K27 acetylation by p300/CBP (histone acetyltransferase p300-EP300 bound to CREB-binding protein CBP-CREBBP) of non-H3K27M mutated H3 protein.^7^ Acetylated H3K27 then preferentially localizes to gene promoters and/or enhancers to activate transcription.

Unfortunately, there are no therapies available that target the H3-alterations that give rise to DMG. However, dordaviprone (also referred to as ONC201 and TIC10) is a first in class, brain penetrant, small molecule of the imipridone family, with reported case study benefits for the treatment of H3K27M mutant DMG,^20^ and in clinical trials following radiation and at disease progression (NCT02525692).^21^ This has encouraged off-patent development,^22,23^ and clinical controversy.^24^ Here, we outline the discovery of dordaviprone for the treatment of DMGs and provide a summary of the known mechanisms of action (MOA) and touch on other therapies that exploit similar MOAs for the potential improved treatment of DMG.

## Discovery of Dordaviprone (ONC201) for the Treatment of DMG

The first report of dordaviprone was by a patent filed in 1973 outlining its chemical structure. While no MOA or biologics were reported, there was speculation of potential clinical utility for the treatment of CNS disorders.^25^ Dordaviprone's potential as an anticancer agent began to be uncovered in 2013, when it was discovered in a small molecule screen of p53-independent inducers of TNF-related apoptosis-inducing ligand (TRAIL) mediated cell death in colorectal cancer cell lines in vitro.^26^ This stimulated interest in its clinical potential due to a lack of therapeutics that are effective against *TP53*-mutant tumors and its potential pan-cancer utility.

During a Phase II clinical trial of dordaviprone in adult glioblastoma patients, using 625 mg administered orally every 3 weeks (NCT02525692), a 22-year-old female patient harboring an H3K27M positive thalamic HGG, confirmed via biopsy, experienced a near complete objective response including regression of the primary thalamic lesion, durable 3-years later.^27^ This led to significant clinical interest in the potential use of dordaviprone in pediatric H3K27M mutant DMG, where H3K27M is the defining feature. Hence, an expanded access program was launched to further examine its therapeutic potential. A preliminary clinical study using dordaviprone in patients harboring DMG or DIPG, post-radiation therapy (RT) and with confirmed signs of radiological progression commenced,employing dordaviprone orally 625 mg per week.^21^ Among the 4 pediatric patients who started dordaviprone post-RT, 2 patients remained in second progression-free survival (2PFS) for 53 and 81 weeks, with several others experiencing radiographic regression after commencement, including 1 patient with a complete response. These findings encouraged numerous other clinical trials to examine dordaviprone in multiple tumor types as a single agent and in combination (Tables 1 and 2).Recently, the combined results of two DMG clinical trials testing dordaviprone (NCT03134131 and NCT03416530) following initial RT but prior to recurrence, demonstrated an increased median OS of 21.7 months compared to historical, while those treated after recurrence had a median OS of 9.3 months (Table 1).* Remarkably, DMG patients who experienced extended benefits from dordaviprone treatment, showed reversed hallmark loss of H3K27me3, identified by IHC upon tissue autopsy, with in vitro data suggesting the mechanism to be through metabolic inhibition of the Jumonji C domain (Jmj C) family of histone lysine demethylases.

**Table 1.** Dordaviprone Clinical Trials in Central Nervous System Cancers

Identifier	Conditions	Date	Phase	Intervention	Description	Published Results
NCT02038699	Advanced Glioblastoma Advanced Colorectal Cancer Advanced Triple-negative Breast Cancer Advanced Non-small Cell Lung Cancer	Jan 2014 to Dec 2016.	Phase 1/2	ONC201	Testing ONC201 in patients with advanced cancer. The Phase 1 portion will inform the dosing and safety profile, while the Phase 2 portion will determine efficacy.	Withdrawn.
NCT02525692	Glioblastoma Diffuse Midline Glioma H3 K27M Glioma Thalamic Glioma Infratentorial Glioma Basal Ganglia Glioma	Jan 2016 to Dec 2023.	Phase 2	ONC201	Testing the efficacy of ONC201 in patients with recurrent glioblastoma or WHO Grade IV gliomas with the H3 K27M mutation.	Among the 14 patients with recurrent disease prior to initiation of ONC201 treatment, median PFS is 14 weeks, and the median OS is 17 weeks. Among the 4 pediatric patients enrolled, 2 DIPG patients remain progression-free for at least 53 and 81 weeks.
NCT03134131	Diffuse Midline Glioma	28 Apr 2017 to 14 Mar 2022.	Expanded access	ONC201	Expanding access to ONC201 in eligible patients with previously treated glioma harboring the H3 K27M mutation and/or in midline high-grade gliomas.	Eleven of 41 patients were enrolled following radiation but prior to recurrence; 30 of 41 patients were enrolled with recurrent disease. Patient survival data was combined with data from NCT03416530, reporting a median OS of non-recurrent H3K27M-DMG patients treated with ONC201 (n=35) of 21.7 months from diagnosis and 9.3 months at recurrence.
NCT03295396	Diffuse Midline Glioma	31 Oct 2017 to 15 May 2023.	Phase 2	ONC201	To determine the efficacy and safety of ONC201 in adult patients with recurrent H3 K27M high-grade glioma.	Active, not recruiting. No results posted.
NCT03416530	Diffuse Intrinsic Pontine Glioma Glioma, Malignant	29 Jan 2018 to 31 May 2023.	Phase 1	ONC201	Multicenter, open-lab, 7 arms, dose escalation study of ONC201 for pediatric patients with newly diagnosed DIPG and recurrent/refractory H3 K27M gliomas.	Twenty-four of 30 patients were enrolled following radiation but prior to recurrence; 6 of 30 patients were enrolled with recurrent disease. Patient survival data was combined with data from NCT03134131, reporting a median OS of patients with non-recurrent H3K27M-DMG treated with ONC201 (n=35) of 21.7 months from diagnosis and 9.3 months at recurrence. The utility of cf-tDNA in both plasma and CSF of DMG patients was shown to be feasible and to harbor clinical utility. H3K27M VAF values in cf-tDNA were a strong biomarker of response.
NCT04617002	Diffuse Midline Glioma H3 K27M	5 Nov 2020 to Feb 2023.	Expanded access	ONC201	An intermediate-size expanded access protocol to provide ONC201 to patients harboring H3 K27M-mutant and/or midline gliomas who cannot access ONC201.	Available. No results posted.
NCT04629209	Glioblastoma	15 Feb 2021 to 30 June 2023.	Phase 2	ONC201	Open-label study testing intra-tumoral concentrations and radiographic efficacy in adults with EGFR-low glioblastoma. Excluding patients with midline gliomas, H3K27M mutations, or IDH mutations.	Withdrawn (change in approach to study). No results posted.
NCT04854044	Recurrent Glioblastoma Recurrent Gliosarcoma Recurrent Supratentorial Glioblastoma Supratentorial Gliosarcoma	1 May 2021 to 1 July 2026.	Phase 1	ONC201 Radiation Therapy	Combines ONC201 with standard-of-care radiotherapy before tumor resection in recurrent glioblastoma (GBM) patients.	Withdrawn (The P.I. is not prepared to move forward at this time).
NCT05009992	Diffuse Intrinsic Pontine Glioma Diffuse Midline Glioma, H3 K27M-Mutant Recurrent Diffuse Intrinsic Pontine Glioma Recurrent Diffuse Midline Glioma, H3 K27M-Mutant Recurrent WHO Grade III Glioma WHO Grade III Glioma	20 Oct 2021 to 30 June 2027.	Phase 2	ONC201 Radiation Therapy Paxalisib	A Phase 2 trial that determines if the combination of ONC201 and paxalisib is effective for treating patients with diffuse midline gliomas (DMGs).	Recruiting. No results posted.
NCT05392374	Diffuse Intrinsic Pontine Glioma	26 May 2022.	Expanded access	ONC201	Intermediate study to provide access for patients with diffuse intrinsic pontine gliomas who cannot access ONC201 through clinical trials.	No longer available, no results posted.
NCT05476939	Diffuse Intrinsic Pontine Glioma Diffuse Midline Glioma, H3 K27M-Mutant	29 Sept 2022 to Sept 2031.	Phase 3	Everolimus ONC201 Radiotherapy	Also known as BIOMEDE 2.0, it is the second stage of a multi-arm, adaptive platform trial. It is a randomized open-label Phase-3 controlled trial evaluating the efficacy of ONC201 in comparison with everolimus and subsequently historical controls.	Recruiting. No results posted.
NCT05580562	H3 K27M Diffuse Midline Gliomas	23 Jan 2023 to Aug 2026.	Phase 3	ONC201 ONC201 + Placebo Placebo	Randomized double-blind, placebo-controlled, parallel-group, international Phase 3 study for patients with newly diagnosed H3 K27M-mutant diffuse midline glioma, combined with frontline radiotherapy. Excludes patients diagnosed with DIPG.	Recruiting. No results posted.

**Table 2.** Dordaviprone Clinical Trials across Non-CNS Cancers

Identifier	Conditions	Date	Phase	Intervention	Description	Published Results
NCT02250781	Solid Tumors	12 Jan 2015 to 25 Oct 2018.	Phase 1	ONC201	Testing the side effects and best dosing for ONC201 in treating patients with advanced solid tumors.	Completed. No results posted.
NCT02324621	Unspecified Adult Solid Tumor	20 Feb 2015 to 23 Oct 2018.	Phase 1	ONC201	To evaluate long-term efficacy of ONC201 in patients with solid tumors that have metastasized for patients who have previously benefitted from the drug.	Completed. Prolonged stable disease for >6 months was observed in 23.8% (5/21) of patients.
NCT02420795	Central Nervous System Lymphoma Gastric Mantle Cell Lymphoma Recurrent Mantle Cell Lymphoma Recurrent Non-Hodgkin Lymphoma Refractory Mantle Cell Lymphoma Refractory Non-Hodgkin Lymphoma Splenic Mantle Cell Lymphoma	3 Nov 2015 to 16 Nov 2020.	Phase 1/2	ONC201	Testing safety and efficacy of ONC201 in treating patients with non-Hodgkin’s lymphoma that is recurrent or refractory.	Terminated (Per PI request). Median progression-free survival on 625 mg ONC201 was a median of 5 weeks.
NCT02392572	Recurrent Acute Lymphoblastic Leukemia Recurrent Acute Myeloid Leukemia Recurrent Myelodysplastic Syndrome Refractory Acute Lymphoblastic Leukemia Refractory Acute Myeloid Leukemia Refractory Myelodysplastic Syndrome	3 Nov 2015 to 30 Nov 2023.	Phase 1/2	ONC201	Testing the safety and dosing of ONC201 in patients with relapsed/refractory acute leukemia or high-risk myelodysplastic syndrome.	Recruiting. No results posted.
NCT02609230	Advanced Solid Tumors Multiple Myeloma	5 Nov 2015 to 26 Mar 2020.	Phase 1	ONC201	Testing the safety and MTD of ONC201 in patients with advanced solid tumors or multiple myeloma.	Completed. No results posted.
NCT02863991	Multiple Myeloma	1 Jan 2016 to 31 Dec 2022.	Phase 1/2	ONC201	Open-label study of ONC201 in combination with dexamethasone for patients with relapsed/refractory multiple myeloma.	Active, not recruiting.
NCT03099499	Endometrial Cancer	8 June 2017 to Sept 2023.	Phase 2	ONC201	Testing the clinical benefit of ONC201 in women with recurrent or metastatic endometrial cancers, especially those with alterations in the PI3K/mTOR pathway.	Suspended due to slow accrual.
NCT03034200	Recurrent Neuroendocrine Tumor Metastatic Neuroendocrine Tumor	2 Aug 2017 to 30 Nov 2022.	Phase 2	ONC201	Testing whether ONC201 has efficacy in tumor and metastases reduction in PC–PG (pheochromocytoma–paraganglioma) and other neuroendocrine tumors.	Completed. The trial was separated into 3 arms; cohorts A (PC-PG) and B (other neuroendocrine tumors) were treated once a week with ONC201, while dosage was twice a week in cohort C. Of the 10 PC-PG patients in cohort A, 5 exhibited a partial response, with 2 additional patients having stable disease for more than 3 months. In arm B, there was 1 partial responder and 2 with stable disease for more than 3 months, from 12 total patients. Patients enrolled in arm C with PC-PG (n=8) showed 1 partial responder and 7 with stable disease at 3 months.
NCT03394027	Triple Negative Breast Cancer Endometrial Cancer Hormone Receptor Positive, HER2 Negative Breast Cancer	17 Jan 2018 to 7 Oct 2021.	Phase 2	ONC201	Testing ONC201 in each disease cohort to see if there is lasting efficacy in shrinking tumors.	No objective responses were achieved, however, 18% (4/22) of patients had prolonged stable disease (>9 cycles).
NCT03485729	Endometrial Cancer Recurrent	21 Mar 2018 to 31 Dec 2022.	Phase 2	ONC201	Two-stage, non-randomized, open label, 2-arm trial of ONC201 in women with metastatic or recurrent Type II endometrial cancer, who have already failed at least 1 prior chemotherapy regimen.	Active, not recruiting. No results posted.
NCT03492138	Multiple Myeloma	26 Mar 2018 to 13 Jan 2020.	Phase 1/2	ONC201 Ixazomib Dexamethasone	Single arm, open-label study for patients with relapsed/refractory multiple myeloma testing ONC201, ixazomib, and dexamethasone.	Terminated due to low enrollment. No results posted.
NCT03733119	Triple Negative Breast Cancer	13 Nov 2018 to 13 Feb 2021.	Phase 2	ONC201 Methionine-Restricted Diet	Combining ONC201 and a methionine-restricted diet for patients with metastatic or unresectable triple-negative breast cancer (TNBC)	Terminated. The trial was terminated due to slow accrual of patients and statistical analysis was not completed.
NCT03932643	Acute Myeloid Leukemia Myelodysplastic Syndromes	30 July 2019 to July 2025.	Phase 1	ONC201	Pilot study of 20 patients with AML/MDS to determine the safety and preliminary efficacy of ONC201 as a maintenance therapy.	Recruiting. No results posted.
NCT03791398	Metastatic Colorectal Cancer	15 Nov 2019 to 5 Aug 2021.	Phase 1b/2	ONC201 625 mg Nivolumab	Single arm, open label, pharmacokinetic, pharmacodynamics, and efficacy study of ONC201 in combination with Opdivo (Nivolumab) in adult patients with metastatic colorectal cancer.	Terminated. 13 patients were enrolled, and all died of disease progression. Median extension of survival was 58 days. The study was terminated due to lack of efficacy.
NCT04055649	Malignant Ovarian Epithelial Tumor Platinum-Resistant Fallopian Tube Carcinoma Platinum-Resistant Ovarian Carcinoma Platinum-Resistant Primary Peritoneal Carcinoma Recurrent Fallopian Tube Carcinoma Recurrent Ovarian Carcinoma Recurrent Primary Peritoneal Carcinoma Refractory Fallopian Tube Carcinoma Refractory Ovarian Carcinoma Refractory Primary Peritoneal Carcinoma	21 Jan 2020 to 27 April 2024.	Phase 2	ONC201 Paclitaxel	An efficacy trial for combining ONC201 and paclitaxel for patients with platinum-resistant epithelial ovarian, fallopian tube, and recurrent/refractory primary peritoneal cancer.	Recruiting. No results posted.
NCT05542407	Endometrial Cancer Metastasis	15 Apr 2023 to 15 Jan 2025.	Phase 1	Atezolizumab ONC201	Testing the combination of ONC201 and atezolizumab in patients in obese and non-obese metastatic/recurrent endometrial cancer.	Not yet recruiting.
NCT05630794	Colorectal adenomatous polyp Colorectal carcinoma Familial adenomatous polyposis Multiple adenomatous polyps	13 May 2023 to 1 Feb 2025.	Phase 1	Dordaviprone (ONC201)	Testing ONC201 in preventing colorectal cancer in preventing colorectal cancer in patients with familial adenomatous polyposis (FAP) or multiple polyps.	Not yet recruiting. No results posted.

## Access to Dordaviprone through Unconventional Means

Dordaviprone is characterized by a unique heterocyclic pharmacophore that produces no stereoisomers, is highly stable, produced via facile reactions, is aqueous, and passively penetrates the blood–brain barrier (BBB). However, the 1973 patented structure of dordaviprone was mistakenly reported as a [4,3-d] linear structure, rather than the [3,4-e] angular structure identified by recent NMR studies.^22,28^ Wagner et al., re-synthesized both the angular and linear isomers and definitively demonstrated the structures of each, confirming that only the angular isomer exhibited the anti-cancer biology associated with dordaviprone.^28^ The rumored clinical response by DMG patients, and the limited opportunities for patients to receive it worldwide, led to ONC201 becoming available via prescription from a German oncologist and dispensed by a local pharmacy (called GsONC201). Capitalizing on “individual healing attempt” (*individueller Heilversuch*), GsONC201 became a medical treatment option in Germany as it deviates from the medical standard, aimed at treating a specific patient.^24^ However, conjecture remained whether this synthesized version of dordaviprone was the reported inactive [4,3-d] linear isomer of the originating compound,^28^ or the active angular isomer. Duchatel et al., investigated GsONC201, donated from families that had purchased it from Germany, using nuclear magnetic resonance (NMR) spectroscopy, it was identified that the compound was the same active [3,4-e] structural isomer used in clinical trials,^22^ and that the German drug showed analogous in vitro and in vivo anti-DMG effects to the genuine dordaviprone provided under a material transfer agreement. Although hundreds of patients have now received GsONC201, data provided by the clinical teams of 28 pontine H3K27M+ DMG patients starting 7.5 months following their diagnosis (median duration), showed a median OS of 18 months, with patients who received re-irradiation in combination with German dordaviprone survived 22 months.^22^ A recent follow-on study compared the survival of 27 DMG patients with confirmed H3-alterations who purchased and received GsONC201 (*n* = 18) compared with patients who did not (*n* = 9), reporting a median (OS) of 19.9 versus 10.9 months, respectively.^23^

## Safety and Tolerability of Dordaviprone

Dordaviprone is widely considered a safe compound. Its safety profile was first published by Allen et al., in 2013, where it was shown that dordaviprone did not alter cell cycle profiles or decrease clonogenic survival of normal fibroblasts at the same doses that are toxic to colorectal cancer cell lines HCT116 (*TP53*^−/−^).^26^ Reversibly, dordaviprone does, however, decrease the proliferation of fibroblasts, but proliferation is restored 24 h after drug removal.^29^ Subsequently, dordaviprone has been tested on normal bone marrow mononuclear cells, lung epithelial cells, astrocytes, and macrophages without any effect on cell viability at relevant doses.^30–32^

The safety profile in vitro is mirrored in vivo, where Sprague–Dawley rats treated with a single dose of dordaviprone, ranging from 0 to 225 mg/kg, showed no mortalities or dose-limiting toxicities (DLTs).^29^ Only the highest dose resulted in signs of toxicity, including decreased activity, body weight gain, and food consumption as well as abnormal gait. However, both decreased activity and abnormal gait quickly resolved. Furthermore, both blood and urine tests as well as tissue examination suggested no significant toxicities nor long-lasting affects due to dordaviprone. Similar results were obtained when treating beagle dogs with equivalent doses of dordaviprone. Beagles treated with high doses (42 and 120 mg/kg) showed symptoms including salivation, vomitus, loose feces as well as decreased activity on day of administration with the symptoms resolving within a day. Both these preliminary studies suggest no observed adverse event level (NOAEL) at the corresponding human dose of approximately 1.25 g.^29^

The first-in-human clinical trial of dordaviprone set out to determine the recommended Phase II dose (RP2D) as well as conducted safety appraisal for dordaviprone delivered using dose escalation regimen starting at 125 mg, and ranging up to 625 mg.^33^ Dordaviprone was given orally every 21 days to patients with advanced solid tumors. No adverse events (AE) > Grade 1 were observed at any dose, with Grade 1 AEs including fever, nausea, and emesis in 1 patient, and increased serum amylase in 2 patients. The mean half-life of dordaviprone was determined to be 11.3 h, with a C_max_ (maximal concentration) of 6.3 µg/mL and T_max_ (the time it takes for a drug to reach C_max_) at 1.8 h after administration.^33^ The mean AUC observed was 37.7 h *×* µg/mL and the mean CL/F was 25.2 l/h.

Following the optimistic safety profile using 625 mg dordaviprone once every 3 weeks, a Phase I study investigated the safety of 375 or 625 mg dordaviprone orally once per week.^34^ Likewise, no AE >Grade 1 or DLTs were reported. Pharmacokinetic (PK) evaluation identified a mean half-life of 9.4 h, C_max_ of 4.3 µg/mL, and AUC of 34.3 h *×* µg/mL, with similar results after the first and fourth dose. More recently, administration of dordaviprone twice a week on 2 consecutive days has been investigated. Similarly, preliminary studies showed no DLTs or serious AE associated with dordaviprone used twice a week.^35^ These data were built upon by Gardner et al., studies, where a Phase 1 trial of pediatric DMG patients administered 625 mg (weight adjusted), identified similar C_max_ (2.3 µg/mL),^36^ however, within the therapeutic ranges reported by Stein et al.^33^ The maximum-tolerated dose (MTD) was not identified, calling for further clinical investigations. To date, the most common drug-related AEs are fatigue, decreased lymphocyte count, nausea, and vomiting.

## Dordaviprone Mechanism of Action: DRD2 or ClpP?

For the most part, DMG clinical trials examined the dordaviprone safety and efficacy profile without the establishment of robust preclinical data for its use, including no in vivo DMG orthotopic xenograft studies, or understanding of its cellular target. The MOA of dordaviprone has been subject to rigorous debate over recent time. Indeed, the initial MOA reported by Allen et al., in colon cancer suggested dordaviprone promotes TRAIL-mediated apoptosis independent of p53, leading to inhibition MAPK/ERK and PI3K/Akt signaling.^26^

## Dordaviprone as a Selective DRD2 Antagonist

Using a Bayesian machine-learning approach, dopamine receptors, specifically dopamine receptor D2 (DRD2), was identified as a targets of dordaviprone in non-DMG cancer cell lines,^37^ with in silico modeling confirming dordaviprone binds to the active site of DRD2.^22^ Further characterization revealed dordaviprone treatment modulated cyclic adenosine monophosphate (cAMP), a measure of DRD2 activation.^37^ DRD2 is a member of the 5 G-protein coupled dopamine receptor family, split into 2 groups: D1-like (DRD1 and DRD5), which induce cAMP production through an α stimulatory subunit (G_α__s_-coupled), and D2-like (DRD2, DRD3, and DRD4), which inhibit cAMP production through an α inhibitory subunit (G_α__i_-coupled).^38^ DRD2 has been reported as highly expressed across brain tumor types, promoting tumor growth, and has emerged as a therapeutic target for gliomas.^21,39^ DRD2 signaling has previously been targeted in glioblastomas using anti-psychotic therapies, such as haloperidol, decreasing downstream ERK signaling and subsequent tumor growth,^40^ however, clinical trial results in non-DMG HGGs have not supported DRD2 antagonism as an MOA in this disease setting.

Neurotransmitter research in the context of psychiatric disorders such as schizophrenia shows that patients using antipsychotics that antagonize DRD2 experience lower incidences of cancer, even though this patient population is usually associated with social behaviors that increase cancer incidences (excessive smoking and alcohol usage).^41^ This provides some support for dordaviprone’s proposed MOA through DRD2 antagonism, as neurotransmitters such as dopamine and serotonin contribute to tumor initiation.^42^ Notably, using a G protein-coupled receptor (GPCR) screen, dordaviprone did not antagonize any other GPCRs/or dopamine receptors and is shown to harbor more potent anti-tumor effects than more traditional DRD2 antagonists, while maintaining a wider therapeutic index.^43^ DRD2 receptors harbor specific binding pockets containing both orthosteric and allosteric residues, both of which dordaviprone can bind to, allowing for tight molecular docking.^22,44^ Initially, dordaviprone received attention due to promising preclinical data showing upregulation of TRAIL, a potent apoptosis-promoting pathway in multiple cancers.^26^ Antagonism of DRD2 has also been shown to reduce cell viability in triple-negative breast cancer (TNBC),^38^ however, in a TRAIL-independent manner.^45^ Intriguingly, evidence in colon cancer cell lines suggest that DRD5 may influence the sensitivity of tumor cells to dordaviprone, as it is a negative regulator of DRD2 antagonism and is associated with reduced sensitivity.^43^

**Figure 1.**
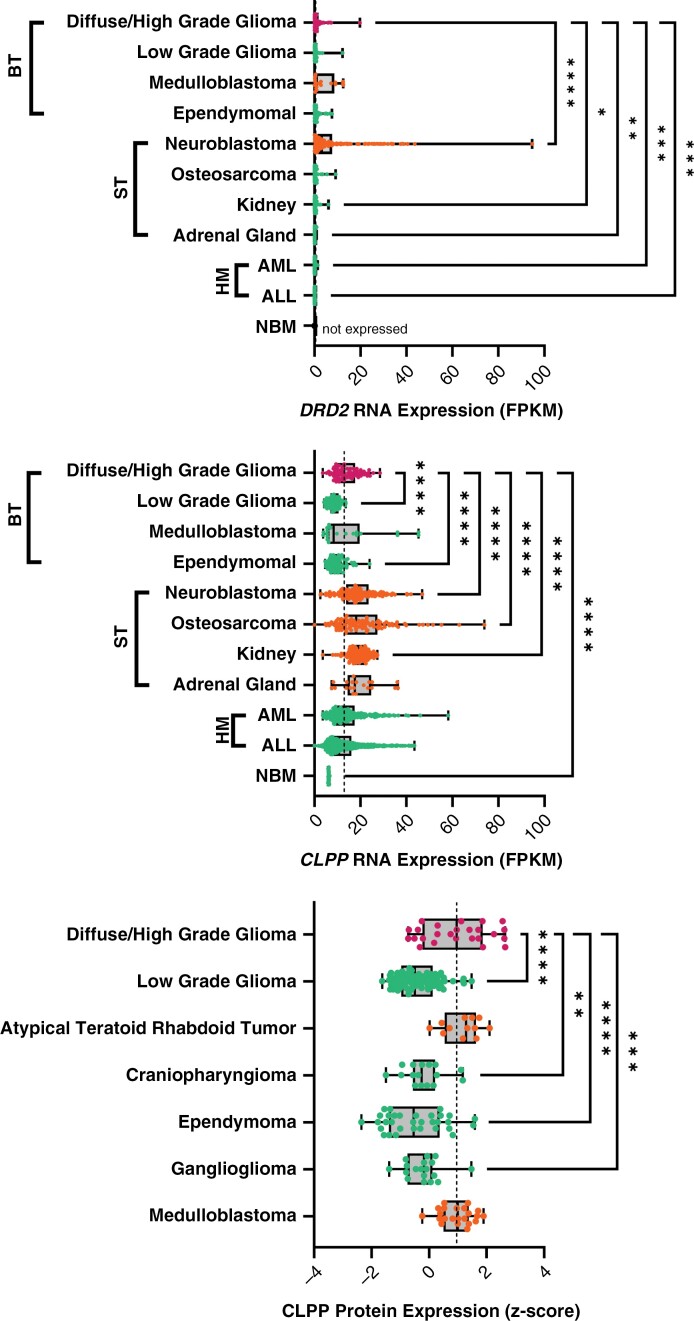
Expression of dordaviprone cellular targets across pediatric cancers, compared with diffuse gliomas. (A) *DRD2* and (B) *CLPP* mRNA expression in diffuse glioma (including diffuse midline glioma, diffuse pontine glioma, and other high-grade gliomas), low-grade glioma, medulloblastoma, ependymoma, neuroblastoma, osteosarcoma, kidney, adrenal gland, AML (acute myeloid leukemia), ALL (acute lymphoid leukemia), and NBM (normal bone marrow). Diseases are grouped into BT (brain tumor), ST (solid tumor), and HM (hematological malignancies). RNA expression data were analyzed from publicly available data (normalized fragments per kilobase of transcript per million mapped reads [FPKM]).^46^ (C) CLPP protein expression (*z*-score) across pediatric central nervous system cancers analyzed from publicly available data.^49^ The dotted line is the median expression level for diffuse glioma, while turquoise dots are less than the dotted line and orange have the median above the dotted line. Statistical significance determined compared only to diffuse gliomas (one-way ANOVA, *P < .05,**P < .01,***P < .001,****P < .0001).

Although there is very little preclinical evidence correlating DRD2 antagonism and response in DMG, there is in silico and in vitro modeling providing evidence that dordaviprone binds to GPCR, in a selective way.^44^ Additionally, there is little information into the interactions of other dopamine receptors, and how these may contribute to its anti-tumor effects. However, mRNA expression does not tend to correlate with dordaviprone sensitivity, in fact, tumor cells with no DRD2 expression have shown sensitivity to dordaviprone (Figure 1A),^46^ suggesting alternative MOAs.^47,48^

## Dordaviprone is an Agonist of the Mitochondria Protease ClpP

**Figure 2.**
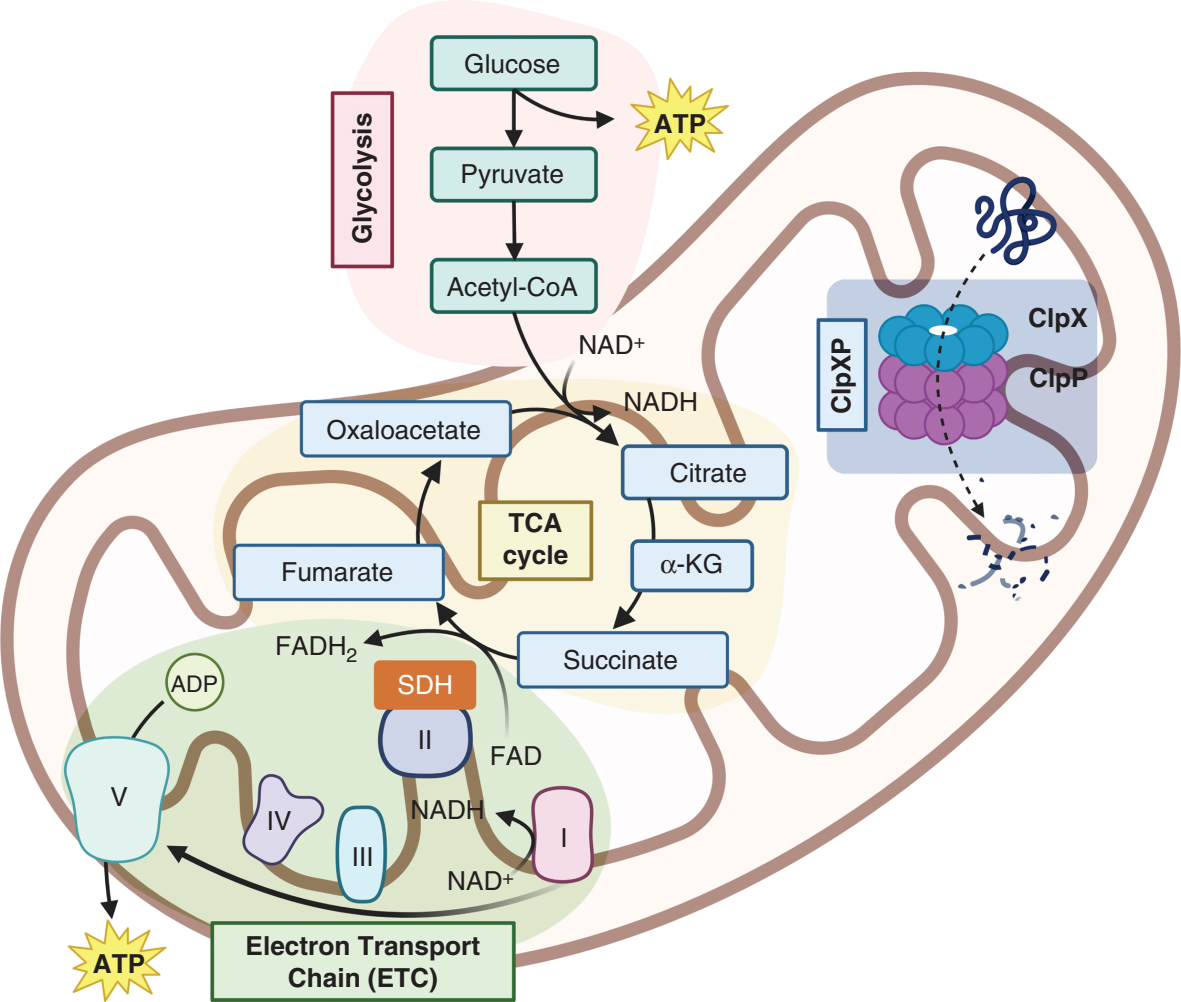
Normal mitochondrial function in cells. ATP production in mitochondria is produced by a cascade of reaction including glycolysis, tricarboxylic acid (TCA) cycle, or electron transport chain (ETC). The ClpXP complex, comprised of ClpP and ClpX, degrades misfolded proteins in the mitochondria, to maintain its functional integrity.

More recent studies show dordaviprone is a potent agonist of the ATP-dependent Clp protease proteolytic subunit (ClpP), a mitochondrial protease that degrades mitochondrial respiratory chain proteins to disrupt energy homeostasis.^50,51^ Huge energy demands are required for rapidly proliferating DMG cells located alone the midline, and are primarily filled by the production of adenosine triphosphate (ATP) by the respiratory chain complexes. Substrates generated from glucose are produced during glycolysis and converted into coenzyme A (acetyl-CoA) which enters the tricarboxylic acid cycle (TCA cycle), to produce nicotinamide adenine dinucleotide + hydrogen (NADH) to generate a proton gradient across the inner mitochondrial membrane (Figure 2).^52,53^

**Figure 3.**
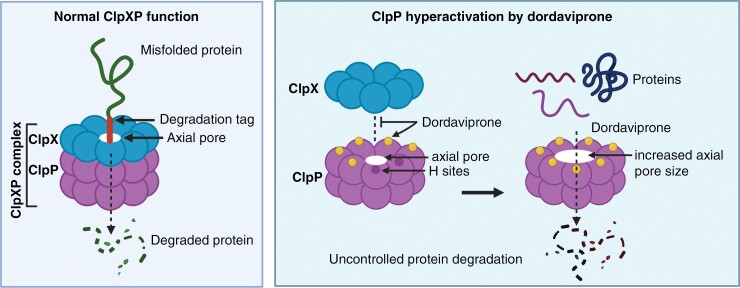
Hyperactivation of ClpP by dordaviprone. During respiration, misfolded proteins are identified by a degradation tag and degraded by the ClpXP complex, passing through the axial pore. Dordaviprone replaces ClpX and binds ClpP, in H sites, removing protein selectivity and hyperactivating its protease activity. Additionally, dordaviprone increases axial pore size, resulting in more efficient degradation of mitochondrial respiratory complex proteins, regardless of their folding.

Mitochondria harbor intricate protein quality control systems, regulated by molecular chaperones which repair misfolded proteins, while proteases, such as the ClpXP complex, degrade misfolded proteins that are beyond repair.^52^ The ClpXP complex is made of 2 subunits; ClpP and ClpX. The ClpP proteins form a chamber-like structure of 2, stacked heptameric rings, creating a hollow core, known as axial pores (Figure 3).^48^ Hydrophobic binding sites (H sites) then act as attachment points for the ClpX protein, activating the complex.^48,52^ ClpX is part of a AAA+ (*A*TPase *A*ssociated with diverse cellular *A*ctivities) chaperone class of proteins, a well-known ATPase used in a range of protein machines.^54^ ClpX acts as a protein quality control system, ensuring that only misfolded proteins are proteolyzed due to the recognition of aromatic residues and specific sequences only visible in misfolded proteins.^50,55^

Given the high demands on energy production to fuel the malignant proliferation of DMG; and the observation that ClpP is overexpressed across diffuse gliomas at an mRNA and protein level (Figure 1B and C),^46,49,56^ degradation of the mitochondria matrix heralds huge anti-tumor potential.^51^ Dordaviprone is able to selectively bind to the H sites of ClpP, displacing ClpX, leading to its degradation as dordaviprone concentration increases.^51^ Upon dordaviprone attachment to the ClpP H sites, axial pores enlarge, allowing for increased protein influx into the complex (Figure 3),^22^ causing ClpP to lose the chaperoning of ClpX, and the ability to identify misfolded proteins and translocate them to the ClpXP matrix for degradation.^51^

Duchatel et al., confirmed ClpP target of dordaviprone using in silico binding of dordaviprone to ClpP, and robust structural stability leading to the degradation of mitochondrial proteins, such as SDHA and SDHB; components of Complex II of the ETC, in DMG cell lines in vitro and in vivo.^22^ This was further supported by Przystal et al., also showing ClpP as a key mediator of the response to dordaviprone in DMG.^51^ These findings were then validated through the use of CRIPSR/Cas-9 mediated knockdown of ClpP in DMG cell lines, where dordaviprone efficacy was lost in cell lines that were previously sensitive.^57^

Broadly, the process whereby dordaviprone binds to ClpP destabilizes the homeostatic levels of proteins in the mitochondria, increasing reactive oxygen species (ROS) and catalyzing the integrated stress response (ISR), an adaptational signaling pathway that allows cells to shutdown protein synthesis.^51,58^ In DMG, dordaviprone has been shown to dramatically increase mitochondrial ROS levels in a time- and dose-dependent manner, leading to mitochondrial structural deformities.^51^ Indeed, studies have reported increased ATF4 (activating transcription factor 4) and CHOP (C/EBP homology protein); known biomarkers for ROS and ISR, to be increased following dordaviprone exposure.^31^ This has been further examined in DMG revealing ATF4, and CHOP to be significantly upregulated following treatment.^22,51^

ATF4 is a regulator of the mitochondrial stress response, driving the translation of cytoprotective genes through activation of the ISR to protect the mitochondria.^59^ ATF4 mediates CHOP response, which is involved in the regulation of genes responsible for proliferation as well as energy metabolism, and has been shown to be an essential factor in the regulation of endoplasmic reticulum (ER) stress-induced apoptosis.^59–61^ Thus, treatments that activate the ISR, such as dordaviprone, drive increased expression of these proteins.^61^ Consequently, tumor cells will undergo mitochondrial degradation-dependent apoptosis, leading to the inability of tumor cells to proliferate and survive.

## ClpP is the Prominent Biomarker of Response to Dordaviprone in DMG

Cancer cells harboring limited expression of DRD2 are also sensitive to dordaviprone, suggestive of alternative MOAs.^50,62^ Bonner et al., conducted a meta-analysis using the publicly available Cancer Dependency Map (DepMap) data,^63^ to show that *CLPP* mRNA expression levels correlated with dordaviprone sensitivity, across non-DMG cancer cell lines.^47^ Furthermore, recent DMG cell line data corroborated this analysis showing cells with high ClpP protein expression had increased sensitivity to dordaviprone in vitro.^56^ DepMap did not correlate *DRD2* mRNA expression with dordaviprone sensitivity,^47^ nor did it at a transcript level in DMG cell lines.^56^ Although, DRD2 protein expression levels in DMG cell lines in vitro correlated with dordaviprone sensitivity, knockout of DRD2 was lethal to all DMG cell lines in vitro, with some of these DMG cells lines refractory to dordaviprone exposure.^56^ Additionally, Kline et al., showed that transient knockdown of DRD2 in colorectal cancer cells minimally impacts response to dordaviprone, suggesting DRD2 may not be critical for its anticancer MOA.^64^

Here, we performed analysis of *DRD2* and *CLPP* mRNA expression levels obtained from publicly available pediatric cancer transcriptomic data sets, established by St. Jude Hospital, to show limited *DRD2* expression across pediatric cancers (Figure 1A). Neuroblastoma (median = 3.099 normalized fragments per kilobase of transcript per million mapped reads [FPKM]) harbored significantly increased expression compared with diffuse gliomas (median = 0.4941, *P ≤* .001), while there was no difference across brain tumors; and as expected, expression in hematological malignancies was significantly lower than diffuse gliomas (AML median = 0.00331, *P =* .0005, ALL median = 0.002202, *P* = .0004).^56^ Given the very low level mRNA expression reported herein (Figure 1A), it is intriguing that molecular inhibition of *DRD2* blocked proliferation of DMG cell lines in vitro, including DMG cell lines that lacked dordaviprone sensitivity.^56^*DRD2* expression levels in DMG and other non-brainstem pediatric HGGs does not appear to be different,^56^ with recent studies in glioblastoma cell lines showing molecular inhibition of *DRD2* to not effect neurosphere formation or proliferation in vitro.^65^ Potentially, the co-occurrence of high protein expression levels of both DRD2 and CLPP correlates with sensitivity to dordaviprone in vitro, whereas the mRNA levels of *DRD2* or *CLPP* do not. Currently, we are unaware whether high-level protein expression of DRD2 and CLPP correlates or co-occurs with dordaviprone sensitivity in non-brainstem pediatric HGG cell lines.

*CLPP* mRNA is highly expressed in diffuse gliomas compared with lower-grade brain tumors, while solid tumors and hematological malignancies were either significantly increased or showed no difference (Figure 1B). More aggressive cancer subtypes tended to express more *CLPP* mRNA than low-grade cancers. Analysis of CLPP protein expression across pediatric brain tumors,^49^ also identified increased expression in diffuse gliomas compared with all other tumor types of the CNS, potentially highlighting its utility as a precision medicine for diffuse gliomas, solid tumors, as well as acute myeloid and lymphoblastic leukemias and hence it is currently in clinical studies in hematological malignancies (Table 1, Figure 1C). Taken together, these data indicate that ClpP is a more robust biomarker of response to dordaviprone rather than DRD2 in diffuse gliomas,^47,56^ with more work required to determine the role of DRD2 in response to dordaviprone in DMG.^56^

## Potential for Combination Treatment Strategies Including Dordaviprone in DMG

To date, there have been 3 different preclinical combination strategies using dordaviprone in DMG; (1) therapies targeting enzymes responsible for epigenetic posttranslational modifications, (2) a kinase inhibitor, and (3) standard of care radiotherapy (RT). The altered chromatin landscape of DMGs are, for the most part, reversible (except for the 3%–17% of H3 peptides harboring the H3K27M mutation). Therefore, drugs targeting epigenetic enzymes have been combined with dordaviprone and have shown to hold some synergistic potential, at least in vitro. Histone deacetylases (HDACs), enzymatically remove acetyl groups, regulating gene expression across many different cancer types. Nguyen et al., showed HDAC inhibition with either panobinostat and romidepsin-induced synthetic lethality when combined with imipridones which suppressed tumor cell metabolism in glioblastoma cells.^66^ Unfortunately, most HDAC inhibitors have poor BBB permeability,^67^ reducing their potential as a treatment for DMG. An additional epigenetic targeting strategy is to inhibit the histone methyltransferase catalytic enzyme EZH2, of the PRC2 complex, which is retained at genes responsible for neurogenesis and differentiation, processes inhibited in DMG.^17,68^ The rationale for their use is to sensitize DMG cells further to dordaviprone as they mimic the H3K27M mutation that has been associated with patient response to dordaviprone.^21^ EZH2 inhibitors EPZ-6438 (tazemetostat), FDA approved for the treatment of follicular lymphoma and epithelial sarcoma, and PF-06821497, currently in clinical trials for multiple cancers (NCT05767905 and NCT03460977) have been combined with dordaviprone in DMG with some in vitro synergistic potential.^48^ This synergy has been shown across several tumor cell lines, most potently in the glioblastoma cell line, U251, with some efficacy in the highly aggressive DMG cell line, SF8628.^48^ Due to the limited in vivo testing of this triple therapy, there is still some way to go before we determine the clinical potential of these therapies for the treatment of DMG.

Dordaviprone targets mitochondrial energy homeostasis in DMG, however, in DMG cell lines harboring reduced sensitivity, redox-activated PI3K/Akt signaling promotes metabolic adaptation.^56^ Using 13 patient-derived cell lines, representative of the molecular landscape of the DMG patient population (ie, 15% EZHIP, 31% H3.1K27M, 54% H3.3K27M), varying levels of sensitivity to dordaviprone were identified, mimicking the patient experience, where patients often fail upfront treatment. Employing a proteogenomic profiling approach, exposure to dordaviprone in DMG cells increased mitochondrial oxidative stress that was seen to promote redox activation of PI3K/Akt signaling, resulting in metabolic adaption and cell survival. Using paxalisib, a brain penetrant PI3K/Akt inhibitor, additive, and synergistic combinations were shown both in vitro and in vivo, with limited toxicities seen in orthotopic models, or in healthy, human peripheral blood mononuclear cell controls. These studies also correlated phosphatidylinositol-4,5-bisphosphate 3-kinase catalytic subunit alpha (*PIK3CA)* mutations (frequently altered DMG gene^5^) with increased sensitivity to dordaviprone. This is likely due to constitutive activation caused by mutations in the catalytic subunit of PI3K/Akt/mTOR signaling; resulting in cells not being able to further upregulate PI3K/Akt signaling in response to treatment, and hence reducing the metabolic adaption necessary for additional growth following ClpP-agonism. Whereas, *TP53* mutations (second most frequently mutated gene in DMGs^5^) were correlated with decreased sensitivity.^56^ Importantly, 2 DMG case studies experienced increased progression-free survival (PFS) using the combination of dordaviprone and paxalisib, with a patient starting the combination soon after the completion of RT demonstrating a dramatic tumor regression and continued PFS, 24 months following diagnosis (continuing). This study provided the impetus to test RT in combination with dordaviprone or paxalisib at diagnosis or progression, or the combination of dordaviprone and paxalisib within 14 weeks of the completion or RT, with each arm supported by a consolidation strategy including dordaviprone and paxalisib in a Phase II clinical trial for DMG/DIPG—NCT05009992 (Table 1).

Many clinical trials combine dordaviprone with RT in patients diagnosed with DMG (Table 1). Focal RT has the potential to reduce tumor volume, extending survival by 2–3 months, while temporarily improving neurological symptoms (~80%).^69^ Once tumors become refractory or throughout disease course, an increasing number of patients receive re-irradiation, supporting a clinical benefit for some.^70^ Gardner et al., examined the tolerability of ONC201 in combination with RT in the Phase 1 clinical trial (NCT03416530) and did not see AE when administered concurrently.^36^ Duchatel et al., correlated the use of re-irradiation in conjunction with dordaviprone, showing that the combination significantly increased median OS compared with patient receiving dordaviprone alone.^22^ Together these data suggest that dordaviprone may be rationally combined with upfront RT.

## Dordaviprone Modulates the Immune Microenvironment

**Figure 4.**
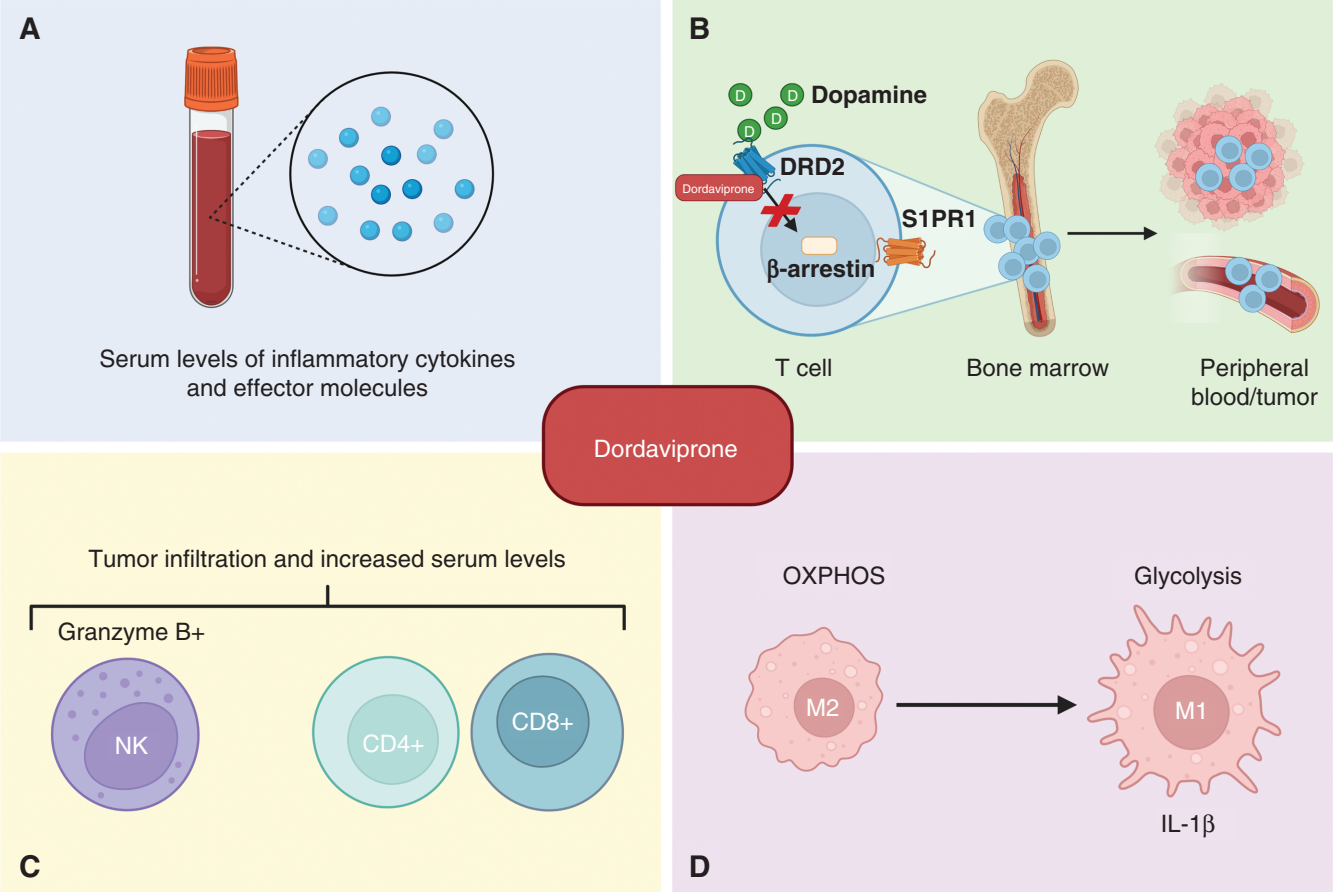
Dordaviprone influences the immune microenvironment. (A) Dordaviprone increases levels of circulating inflammatory cytokines and effector molecules. (B) Proposed mechanism of dordaviprone-induced release of sequestered T cells in the bone marrow. Dordaviprone antagonizes DRD2 signaling, preventing β-arrestin from internalizing S1PR1. S1PR1 surface localization promotes T-cell trafficking. (C) Dordaviprone increases CD4+ and CD8+ T cell levels, as well as NK cells both in the peripheral blood and in the tumor microenvironment of solid tumors, (D) to reprogram macrophage metabolism driving the proinflammatory M1 phenotype.

Emerging data suggests that dordaviprone might not solely work by targeting cancer cells directly, but also by initiating an immune response (Figure 4).^71^ Indeed, several types of immune cells express DRD2 and TRAIL.^72,73^ Preclinical studies using immune-competent mice engrafted with the colorectal cancer lines MC38 and CT126 identified increased numbers of both T cells (CD4 and CD8) and Natural Killer (NK) cells following dordaviprone treatment, within the tumor microenvironment (TME) and spleen as well as in the peripheral circulation.^74^ T cells and NK cells were upregulated in the blood of tumor naïve mice as well, suggesting dordaviprone to directly influence the immune cell populations, and not exclusively through a tumor-immune interaction. Furthermore, tumor cells resistant to dordaviprone in vitro became sensitized in vivo, hypothesizing that dordaviprone may work by activating an immune response against cancer cells. NK cell tumor infiltration was also observed in the breast cancer murine model, E0771, following dordaviprone treatment.^75^ Furthermore, pretreatment of breast cancer cells with dordaviprone prior to the addition of NK cells in vitro, resulted in more efficient killing, suggesting that dordaviprone sensitizes cancer cells to an immune response. Supporting these preclinical results, a tumor tissue biopsy from a patient with metastatic prostate cancer post dordaviprone treatment, showed tumor infiltration of granzyme B+ NK cells.^34^ Additionally, prostate cancer patients showed increased levels of NK cells in the peripheral blood up to 3 days after dordaviprone treatment.^74^

Increased levels of CD45+ immune cells as well as CD8+ T cells were identified in a patient with mantle cell lymphoma 6 months after cessation of dordaviprone treatment (7 cycles of 125 mg every 3 weeks).^76^ However, no NK cells were detected, with authors hypothesizing that this might be due to NK cells being part of the rapid innate immune response and thus no longer detectable after 6 months. However, more definitive studies are required to confirm this finding. Further evidence for an immunomodulatory role for dordaviprone, showed that treatment increased serum levels of both cytokines and effector molecules, including IL-17A, TNF-α, IL-6, IL-10, Granzyme A and B, and perforin in adult patients with solid tumors refractory to standard treatments compared with pretreatment, especially in those who experienced >12 weeks PFS.

Macrophages have also been shown to be modulated by dordaviprone treatment. Primary human monocyte-derived macrophages were shown to switch from respiration to glycolytic ATP production after dordaviprone treatment, in the absence of tumor cells, likely due to mitochondrial stress.^30^ Glycolysis in macrophages has been linked to the proinflammatory subtype M1,^77^ suggesting that dordaviprone drives proinflammatory reprogramming of macrophages. Indeed, macrophages significantly upregulated production and secretion of IL-1β, as well as exhibited a dose-dependent trend of increased TNF although not significant.^30^ However, when co-culturing macrophages with glioblastoma cells in the presence of dordaviprone, no additional decrease in cell viability was observed compared with dordaviprone alone. Only minor changes in expression of inflammatory markers including IL-β1 was seen. This highlights the importance of immune and cancer cell interactions, a field becoming more important as we progress, raising the question as to whether the lack of effect in a co-culture system was due to the immunosuppressive crosstalk between the glioblastoma cells and the immune cells. Indeed, further studies are required to determine these cell-to-cell interactions. It remains to be determined if this could be a result of dosing and/or timing of treatment, macrophages being isolated from other immune cells, including T cells, or the lack of parenchyma/stroma, and how the lack of interactions affects response and will play out in the pons of patients with DMG.

Thus far, no published studies have investigated the immunomodulatory effects of dordaviprone in DMG, however, the potential proinflammatory effects of dordaviprone are promising. DMGs harbor a “cold” TME, characterized by few numbers of tumor-infiltrating lymphocytes as well as inflammatory factors.^6,68,78,79^ Due to a lack of lymphocytes as well as immune checkpoint proteins, immunotherapies including immune checkpoint inhibitors (ICIs) have thus far failed to increase survival in DMG patients, highlighting the need for therapeutics that help to recruit immune cells into the TME.

Dordaviprone’s potential role in the promotion of immune cell infiltration into the TME suggests it might be effectively combined with ICIs. Importantly, dordaviprone increases levels of both NK cells and T cells, both expressing PD-1, making the blockade of PD-1 an attractive approach. Indeed, combining 100 mg/kg dordaviprone with PD-1 inhibitors decreased tumor volume of mice orthotopically engrafted with the undifferentiated colon carcinoma cell line CT26, compared with PD-1 inhibitors alone.^74^ However, the effect was not seen with lower doses of dordaviprone or in mice with chemically induced murine colon adenocarcinoma cells MC38 pressing the need for DMG studies investigating the efficacy of combining dordaviprone with ICIs.

Even though there is evidence for dordaviprone-induced immune cell recruitment, the mechanism underpinning dordaviprone’s immunomodulatory effects remains to be determined. Like DMG, glioblastoma patients harbor a cold TME. Chongsathidkiet et al., identified that both glioblastoma patients as well as glioblastoma immune competent murine glioma models suffer from T cell lymphopenia, specifically CD4+ cells that likely contribute to the cold TME.^80^ The T cells were shown to be sequestered in the bone marrow due to loss of surface expression of the sphingosine 1 phosphate receptor 1 (S1PR1), required for T cells to travel between tissues. This loss of S1PR1 is likely mediated through β-arrestin, downstream of DRD2, suggesting a potential mechanism of dordaviprone-induced immune cell modulation, *the systemic antagonism of DRD2*. Intriguingly, the sequestration of T cells seems to be due to tumor location. When engrafting the same tumor cells either intracranially or subcutaneously, only the intracranial tumors resulted in T cell sequestration. Furthermore, systemic immune suppression has also been observed in patients with traumatic brain injury, identifying a strong-communication link between the CNS and the immune system.^81^ The mechanism underpinning these observation remains to be elucidated; however, β-arrestin/S1PR1 mediated T-cell sequestration and dordaviprone’s role in immunomodulation may suggest dopaminergic signaling might play a yet to be determined but significant role.

## Next-generation ClpP Agonists

The promising clinical benefits testing dordaviprone in clinical trials, expanded access schemes and through unorthodox routes, coupled with the identification of *CLPP* overexpression in aggressive cancers including DMG, has stimulated the development of new ClpP agonists called “TR” analogs. These compounds do not cross-react with, or bind to DRD2, and hence are pure ClpP-agonists.^52,57,62,82^

TR compounds differ from imipridones by substitutions of small lipophilic moieties, combined with optimized benzyl residues (Table 3),^83^ resulting in increased specificity for ClpP and hence amplified potency.^62^ Further refinement of the compound by the removal of the carbonyl oxygen and methyl residue, increased shape complementarity, and optimized noncovalent interactions, resulting in the latest of the TR ClpP-agonists, called “TR-107”; shown to promote ClpP activity and drive anticancer responses in the low nanomolar range.^52,57^ Cells harboring *CLPP* knockout, treated with TR chemicals did not show sensitivity or increased ATF4 activity, demonstrating the ClpP selectivity of these compounds.^82^ Treatment of TNBC cell lines with TR-107 promoted ClpP-mediated degradation of mitochondrial TCA cycle proteins in a dose-dependent manner, however, did not promote a TRAIL-mediated apoptotic response, rather caused cancer cell cytostasis.^57,82^ This suggests that TR compounds may need to be combined with RT, or targeted therapies like paxalisib to show a response in DMG.^56^

**Table 3.** Predicted Brain Penetration Potential of ClpP-agonists as Determined by CNS-MPO.

Name	ONC201		ONC206		TR-27		TR-57		TR-65		TR-107	
Chemical structure	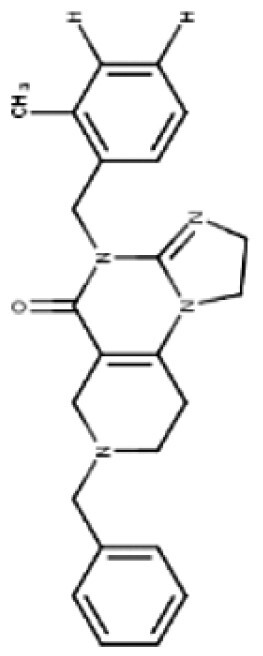		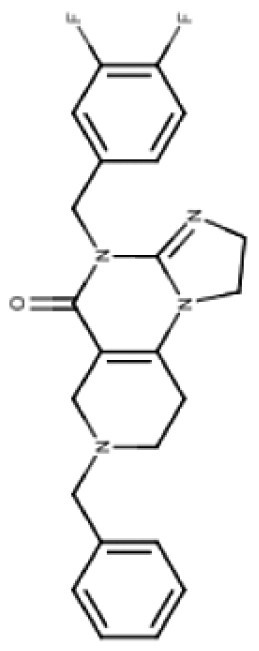		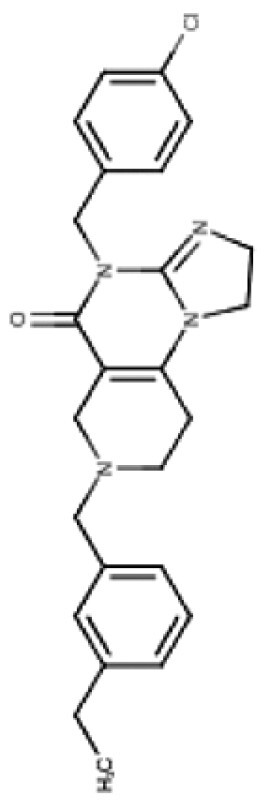		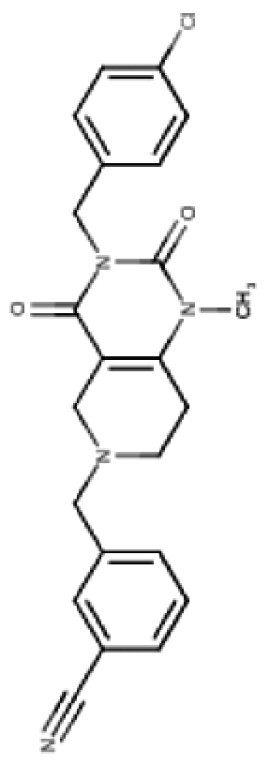		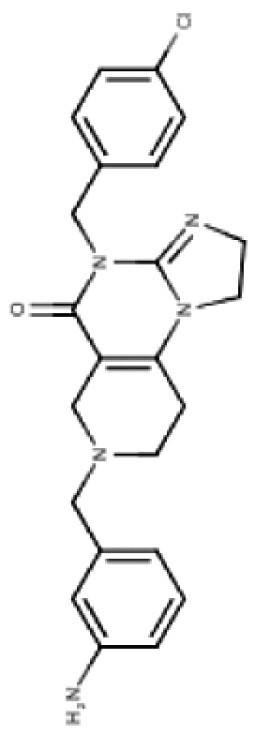		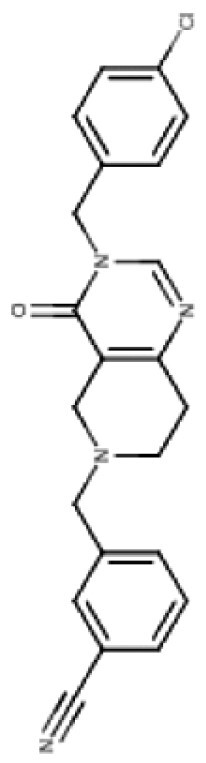	
CNS-MPO Parameter	Actual	Score	Actual	Score	Actual	Score	Actual	Score	Actual	Score	Actual	Score
LOGP	3.05	0.97	2.83	1.00	4.10	0.45	3.06	0.97	2.32	1.00	3.13	0.93
LOGD	2.24	0.88	2.03	0.98	3.27	0.36	3.68	0.66	1.42	1.00	2.78	0.61
MW	386.50	0.81	408.45	0.65	434.97	0.46	420.90	0.57	421.93	0.56	390.87	0.78
TPSA	39.15	0.96	39.15	0.96	39.15	0.96	67.65	1.00	65.17	1.00	59.70	1.00
HBD	0	1.00	0.00	1.00	0.00	1.00	0.00	1.00	2.00	0.50	0.00	1.00
PKA	8.13	0.93	8.12	0.94	8.16	0.92	7.54	1.00	8.23	0.88	7.49	1.00
CNS-MPO Score	5.55/6		5.54/6		4.16/6		5.19/6		4.94/6		5.32/6	

Subjecting imipridones and TR-compounds to analysis using the central nervous system multi-parameter optimization (CNS-MPO) computation tool,^84^ predicted all ClpP-agonists as good candidates to cross the BBB and enter the brain/tumor when delivered systemically (Table 3). CNS-MPO predicts ONC201 and ONC206 to have the best CNS penetration, followed by TR-107 and TR-57, however, at this time, there are no reports of preclinical or clinical examination of TR-compounds in DMG.

## Discussion

Emerging clinical data suggests that some DMG patients treated with dordaviprone experience tumor regression and neurological improvement, and in some cases, increased median OS.^20–23^ These early phase trial results and alternative access opportunities have encouraged phase III efficacy testing in patients diagnosed with DMG, H3 K27-altered located along the midline structures of the brain, but excluding patients harboring tumors located in the pontine region of the brainstem (NCT05580562, Table 1). Potentially, patients with midline/thalamic DMGs show an improved response to dordaviprone, compared with those patients diagnosed with pontine tumors. This is likely due to differing immune cell populations in the TME of DIPG compared with thalamic DMG, where monocyte-derived macrophages are the dominant immune cell population, while DIPGs are enriched in brain-resident microglia.^68^ So not only does this promote different immune responses to the drug, but these immune cell populations promote a mesenchymal cell state for patients with thalamic lesions, whereas DIPGs exhibit stemness properties including oligodendrocyte precursor cell (OPC)-like capable of self-renewal.^68,85^

The brain’s high oxygen demand,^86,87^ pinpoints oxidative phosphorylation, and hence the mitochondria, as an essential factor in the energy supply needed for the rapid cellular proliferation of DMG. Thus, DMGs require abundant respiratory chain proteins to meet these high energy demands. It is therefore clear that this underpins a metabolic dependency in DMG, and hence a therapeutic vulnerability that dordaviprone may exploit. Assessment of *CLPP* gene and protein show high expression in diffuse gliomas and other aggressive pediatric cancers (Figure 1B and C), highlighting the clinical potential of ClpP agonists.^51,56^ Development of new selective ClpP agonists, including the TR compounds, may add additional weapons to the barren armory of options we currently have for the treatment of DMG.

Dordaviprone’s excellent safety profile makes it an ideal candidate for the combination strategies that are necessary if we are to achieve long-term positive outcomes, particularly for children diagnosed with DIPG. Hence, dordaviprone is currently being tested in combination with RT and paxalisib in the international clinical trial “Combination Therapy for the Treatment of Diffuse Midline Gliomas” (NCT05009992), underpinned by extensive preclinical and case study results established across 8 international laboratories and coordinated by the Pacific Neuro-Oncology Consortium (PNOC022).^56^ Although we are still some way from achieving long-term survival, dordaviprone appears to be one of the options needed for the future development of complex multimodal combination strategies that will improve outcomes for children, adolescents, and young adults diagnosed with DMG, H3 K27-altered.
